# Endothelial Collapse as a Convergent Immunovascular Phenotype in Sepsis and Severe Arboviral Disease: The SIMVAC Model

**DOI:** 10.3390/pathogens15090952

**Published:** 2026-09-08

**Authors:** Jhan S. Saavedra-Torres, H. A. Nati-Castillo, Alice Gaibor-Pazmino, Marlon Rojas-Cadena, Valeria Estefania Galecio Tito, Kenny Ruiz Sosa, Leonardo Sánchez Salazar, Jefferson A. Núñez-Medina, Juan S. Izquierdo-Condoy

**Affiliations:** 1 Grupo de Investigación Biomedicina, Fundación Universitaria Vision de las Americas, Pereira 660001, Colombia; 2 Facultad de Ciencias de la Salud, Universidad CES, Medellin 050021, Colombia; 3 One Health Research Group, Universidad de las Américas, Quito 170124, Ecuadormarlonrojas1295@gmail.com (M.R.-C.);; 4 Departamento de Medicina Interna, Hospital de Especialidades Eugenio Espejo, Quito 170403, Ecuador

**Keywords:** arboviruses, sepsis, endothelial dysfunction, capillary leak, Severe Infection-Mediated Vascular Activation and Collapse (SIMVAC)

## Abstract

**Background/Objectives:** Severe bacterial sepsis and arboviral diseases may present with overlapping manifestations of vascular leakage, hemodynamic instability, thrombocytopenia, and organ dysfunction. We examined whether etiologically distinct severe infections may converge on selected components of immunovascular dysfunction and propose the Severe Infection-Mediated Vascular Activation and Collapse (SIMVAC) model as a hypothesis-generating framework. **Methods:** This narrative mechanistic review integrates human clinical and physiological studies, translational biomarker evidence, experimental models, and pathogen-specific mechanistic studies, with particular attention to the relative strength of evidence across arboviruses. **Results:** Dengue provides the strongest arboviral mechanistic support, linking plasma leakage with NS1-mediated endothelial barrier disruption, glycocalyx degradation, tissue-specific vascular effects, and inflammatory amplification. Evidence for yellow fever, West Nile virus, and Zika supports distinct organ-specific or systemic pathways but is less complete, whereas evidence for Mayaro, Oropouche, Venezuelan equine encephalitis, and Rocio viruses remains limited or hypothesis-generating. Candidate points of convergence include glycocalyx injury, Ang-2/Tie2 and S1P dysregulation, complement activation, NETosis, and vWF/ADAMTS13 imbalance. **Conclusions:** SIMVAC should be regarded as a testable conceptual model rather than a validated syndrome or diagnostic tool. Prospective, pathogen-confirmed studies are required to determine whether reproducible immunovascular signatures exist across severe infections and whether they provide clinically meaningful information beyond conventional etiological and severity assessment.

## 1. Introduction

Sepsis and severe arboviral infections clinically converge in states characterized by fever, hypotension, impaired tissue perfusion, and multiorgan dysfunction, particularly in tropical and subtropical regions where these etiologies circulate simultaneously [1,2,3]. This phenotypic overlap reduces the ability to achieve early etiological discrimination during the initial hours of care, when decisions regarding antimicrobial therapy, fluid resuscitation, vasopressor support, and hemodynamic monitoring frequently precede microbiological or molecular confirmation [1,4,5]. Clinical similarity, however, does not establish pathophysiological equivalence. The central question is whether distinct infectious stimuli can produce comparable circulatory states through disruption of shared functional nodes within the immunovascular system [1,6,7,8].

The accumulated evidence from dengue provides a mechanistic framework for examining this hypothesis. Classical studies of dengue shock syndrome identified plasma extravasation resulting from a transient increase in vascular permeability as a major determinant of intravascular volume depletion, distinguishing this process from hemorrhagic blood loss as the predominant mechanism [9]. Decades later, studies of dengue virus non-structural protein 1 linked this vascular phenotype to defined molecular mechanisms. Experimental evidence demonstrated that this protein induces hyperpermeability through pathways integrating immune activation with direct disruption of the endothelial barrier [6,10,11]. Activation of sialidases, cathepsin L, and heparanase promotes glycocalyx degradation, loss of sialic acid and heparan sulfate, and subsequent impairment of vascular integrity [6,10]. Thus, a pathogen-specific viral determinant can be translated into functional disruption of the blood tissue interface.

Bacterial sepsis partially converges on this same functional node through a distinct molecular architecture. Microbe derived and damage-associated molecular patterns, inflammatory cytokines, complement, coagulation pathways, platelets, and immune cells interact with the endothelium and modify its barrier, signaling, and vascular regulatory functions [1,7,8]. Glycocalyx degradation, increased vascular permeability, endothelial activation, microcirculatory abnormalities, and loss of perfusion regulation therefore represent points of functional convergence without requiring identity among the molecular pathways through which these alterations arise [7,8].

This distinction between etiological heterogeneity and functional convergence provides the conceptual foundation for the proposed model. Available evidence does not support classifying sepsis and severe arboviral infections as a single pathophysiological entity [12]. Rather, specific disease trajectories can converge on a shared immunovascular network despite originating from distinct pathogen-specific and host-response mechanisms [13]. The coexistence of bacterial infections, different arboviruses, and coinfections further introduces combinations of immune, endothelial, and hemostatic signals whose interactions can modify disease trajectories and the resulting clinical phenotype [2].

In this narrative mechanistic review, the available evidence is interpreted according to source type—human clinical or physiological studies, translational biomarker studies, experimental models, or mechanistic inference—and according to the consistency of pathogen-specific findings. This evidentiary hierarchy is summarized below and is intended to prevent mechanisms established primarily in dengue or bacterial sepsis from being extrapolated indiscriminately to less-characterized arboviruses.

## 2. Dengue as the Mechanistic Anchor for Immunovascular Convergence

Severe dengue provides the strongest arboviral mechanistic basis for evaluating the SIMVAC hypothesis. Historical physiological studies established that dengue shock is characterized by increased vascular permeability and plasma extravasation, producing intravascular volume depletion rather than shock attributable primarily to hemorrhagic blood loss [9]. These observations provided an early physiological description of the vascular component of severe dengue [9].

Subsequent experimental work provided molecular resolution of this phenotype. Dengue virus non-structural protein 1 (NS1) has been shown to disrupt endothelial barrier integrity and promote vascular hyperpermeability [10,11]. NS1-induced activation of sialidases, cathepsin L, and heparanase can degrade components of the endothelial glycocalyx, including sialic acid and heparan sulfate [10], while inflammatory signaling, including reported Toll-like receptor 4-dependent pathways, may further modify endothelial function [11]. Together, these studies connect a pathogen-specific viral determinant with structural and functional disruption of the vascular interface [10,11].

Importantly, these findings do not imply uniform vascular biology across flaviviruses. Work from the Harris laboratory and collaborators has demonstrated tissue-specific endothelial effects of flaviviral NS1 [14] and has further linked NS1-mediated endothelial dysfunction with viral dissemination [15]. These observations indicate that endothelial responses depend on both viral identity and vascular bed [14,15]. Inflammasome activation by dengue NS1 provides an additional example of this complexity because inflammatory pathways may contribute to antiviral protection while simultaneously participating in broader tissue responses [16]. Complementing these mechanistic studies, long-term prospective cohort investigations in Managua have demonstrated how pre-existing arboviral immunity and epidemic context can modify subsequent infection and clinical expression, including interactions between prior dengue exposure and symptomatic Zika and differences in transmission and disease severity across successive chikungunya waves [17,18]. Thus, dengue provides a mechanistic proof of principle for pathogen-driven endothelial dysfunction, whereas extrapolation of the same molecular sequence to other arboviruses requires pathogen-specific evidence [10,14,16].

## 3. Epidemiological Context and Heterogeneous Severe Phenotypes

The geographic expansion and cocirculation of arboviruses increase the number of clinical settings in which severe viral infections must be differentiated from bacterial sepsis [19,20]. Climate, urbanization, human mobility, vector adaptation, and population immunity influence the distribution and intensity of arboviral transmission, increasing the probability that multiple etiologies coexist within the same epidemiological setting [3,20,21].

This epidemiological overlap should not be interpreted as biological uniformity. Dengue may progress to plasma leakage, thrombocytopenia, shock, and organ dysfunction [4,9,10,11]; West Nile virus predominantly causes severe disease through neuroinvasion [22]; Zika virus is distinguished by neurotropism, vertical transmission, and uncommon neurological or severe systemic complications [4,23,24]; chikungunya is primarily arthritogenic and may result in persistent joint manifestations [4,25]; and yellow fever is dominated by hepatocellular injury, metabolic dysfunction, coagulopathy, and systemic deterioration, with emerging evidence also supporting endothelial involvement in severe disease [4,26]. These differences reflect pathogen-specific tissue tropism, immune history, age, comorbidities, and host responses [22,24,26].

The relevance of this heterogeneity to SIMVAC is therefore syndromic rather than taxonomic. Distinct infections may present with overlapping combinations of fever, hypotension, thrombocytopenia, altered tissue perfusion, vascular permeability, and organ dysfunction before etiological confirmation, while reaching these clinical states through different upstream mechanisms [3,4,11,24,26]. The framework examined in this review concerns possible convergence at selected endothelial and microcirculatory functional nodes rather than a uniform pathophysiology of arboviral disease [7,10,26,27,28,29].

## 4. Mechanisms of Immunovascular Convergence

The hypothesis of a convergent immunovascular phenotype between sepsis and severe arboviral infections can be examined through mechanisms that, although activated by distinct infectious triggers, may ultimately compromise shared vascular functions [30,31]. This convergence does not imply pathophysiological equivalence between bacterial sepsis, dengue, and other arboviral infections [7,8,32]. Rather, the comparative framework resides in the progressive loss of endothelial homeostasis across interconnected systems regulating inflammation, vascular permeability, hemostasis, and microcirculatory function [8,32]. Within this framework, the angiopoietin-2 (Ang-2)/Tie2 axis, sphingosine-1-phosphate (S1P) signaling, complement activation [30,31], neutrophil extracellular trap formation (NETosis), and von Willebrand factor (vWF)/ADAMTS13 dysregulation provide complementary candidate pathways through which severe infectious insults may converge on endothelial barrier disruption, microvascular instability, and organ dysfunction. Their inclusion in the SIMVAC framework is proportional to the strength of pathogen-specific evidence and does not imply that each pathway is present or equally relevant in every severe infection [30,31,33,34].

The angiopoietin-2 (Ang-2)/Tie2 axis represents an early transition point between vascular stability and endothelial dysfunction [31]. Under homeostatic conditions, angiopoietin-1 (Ang-1) activates Tie2 signaling and maintains endothelial quiescence by preserving intercellular junctions and restricting vascular permeability. During inflammatory activation, Ang-2 is released from Weibel–Palade bodies and shifts this regulatory balance away from endothelial-stabilizing signaling [35,36]. The increased Ang-2/Ang-1 ratio reported in severe infections therefore has functional significance, reflecting progressive loss of the endothelial capacity to maintain vascular barrier integrity. Its association with disease severity and mortality in sepsis further supports evaluation of this axis not only as a biomarker of endothelial injury but also as a candidate translational marker of endothelial destabilization requiring prospective validation across pathogen-defined cohorts [37].

Sphingosine-1-phosphate (S1P) represents a counter-regulatory component of this process. Transported predominantly by apolipoprotein M (ApoM) within high-density lipoprotein particles, S1P contributes to the stabilization of endothelial junctions and limits pathological increases in vascular permeability [38,39]. Reduced S1P availability during severe infectious diseases may therefore diminish this protective signaling precisely when inflammatory and endothelial-destabilizing pathways are amplified. Accordingly, the combination of elevated angiopoietin-2 and reduced S1P may capture the immunovascular transition more comprehensively than either mediator considered in isolation: a signal associated with endothelial destabilization increases while a barrier-protective pathway simultaneously declines [7,37]. This reciprocal imbalance provides a mechanistic framework linking systemic inflammatory activation to progressive loss of endothelial barrier integrity and subsequent vascular dysfunction [30,31,33,34].

The complement system may act as an amplifier of immunovascular dysfunction during severe infection [40,41]. In sepsis, excessive complement activation, particularly C5a signaling, has been associated with dysregulated inflammatory responses and multiorgan dysfunction [40]. In severe dengue, NS1-associated complement activation and increased concentrations of C5a and terminal complement complexes have been linked to vascular leakage [41]. Complement-related abnormalities may therefore coexist with glycocalyx injury and other forms of endothelial dysfunction described in severe dengue and septic shock [7,10,29,41]. Within the SIMVAC framework, complement should consequently be considered a context-dependent amplifier of endothelial and inflammatory dysfunction rather than an independent defining mechanism [40,41].

Neutrophil extracellular trap formation (NETosis) extends this interaction into microcirculation. Neutrophil extracellular traps (NETs) contribute to pathogen immobilization and containment; therefore, their formation does not inherently represent a pathological process [42,43]. However, when NET production becomes excessive or inadequately resolved, extracellular chromatin and associated proteins can promote endothelial injury, platelet activation, and microthrombus formation. This response-dependent transition is relevant to the SIMVAC framework. The pathological determinant is not NETosis itself, but the loss of proportionality between its antimicrobial function and its vascular consequences [42,44]. Under these conditions, an initially protective component of innate immunity can become an amplifier of endothelial dysfunction, immunothrombosis, and microcirculatory impairment, thereby linking dysregulated neutrophil responses to progressive vascular and tissue injury [8,28,30,31,33,42,43,45].

The von Willebrand factor (vWF)/ADAMTS13 axis ultimately links endothelial activation to hemostatic dysregulation. vWF, stored within Weibel–Palade bodies, is released following endothelial activation and promotes platelet adhesion at sites of vascular injury [46,47]. Under physiological conditions, ADAMTS13 limits vWF activity by cleaving ultra-large vWF multimers into less thrombogenic forms. When endothelial vWF release exceeds this regulatory capacity, highly thrombogenic multimers accumulate, promoting platelet adhesion, platelet consumption, and microvascular disturbances [46,47,48]. This relationship provides a mechanistic framework for integrating clinical phenomena that may appear paradoxical: thrombocytopenia and bleeding tendency can coexist with platelet activation, immunothrombosis, and microthrombotic events. Thus, disruption of the vWF/ADAMTS13 balance represents an interface through which endothelial dysfunction can be translated into altered hemostasis and microcirculatory injury [7,46,48].

These mechanisms acquire greater biological meaning when interpreted as an integrated functional network. Glycocalyx degradation compromises the initial protective interface of the vascular surface; angiopoietin-2 (Ang-2)/Tie2 dysregulation promotes endothelial destabilization; reduced sphingosine-1-phosphate (S1P) signaling weakens compensatory barrier-protective mechanisms [49,50,51]; complement activation amplifies inflammation and vascular permeability; neutrophil extracellular trap formation (NETosis) links innate immunity to microvascular injury; and von Willebrand factor (vWF)/ADAMTS13 imbalance translates endothelial activation into hemostatic dysregulation [49,50]. The resulting pathophysiological state is not necessarily identical in sepsis and severe arboviral infections. Rather, distinct upstream mechanisms may converge functionally on a shared vascular phenotype characterized by increased permeability, microcirculatory heterogeneity, impaired tissue perfusion, and progressive organ dysfunction [8,30,31,33,34,45].

## 5. Scope, Operational Definition, and Testability of the SIMVAC Framework

The SIMVAC model is proposed as a hypothesis-generating framework of partial immunovascular convergence rather than as a newly established syndrome, diagnostic category, or clinical decision rule. Its central proposition is that etiologically distinct severe infections may disrupt partially shared vascular functions—including endothelial barrier integrity, vascular regulatory signaling, hemostasis, and microcirculatory perfusion—without sharing identical initiating mechanisms [7,10,26,27,28,29].

No validated operational definition currently identifies a patient as having SIMVAC. An isolated increase or decrease in syndecan-1, angiopoietin-2, sphingosine-1-phosphate, complement mediators, NET-associated markers, von Willebrand factor, ADAMTS13, or other candidate biomarkers cannot establish the construct. Similarly, no SIMVAC-specific biomarker thresholds, sensitivity or specificity estimates, exclusion criteria, or validated composite panels are currently available. Prospective studies should therefore determine whether coordinated abnormalities across endothelial barrier integrity, vascular regulatory signaling, inflammatory amplification, hemostasis, and microcirculatory function define a reproducible biological state.

Important biological differences between bacterial sepsis and arboviral infections must remain explicit within this framework. Bacterial sepsis can involve profound vascular dysregulation, endothelial injury, coagulation abnormalities, and heterogeneous trajectories of organ and microcirculatory dysfunction [5,7]. Arboviral diseases follow pathogen-specific temporal patterns determined by viral replication, viremia, pre-existing immunity, tissue tropism, and immune responses [9,11,22,23,24,25,26]. Severe dengue is characterized particularly by a phase of increased vascular permeability and intravascular volume redistribution [4,9,10,11], whereas yellow fever may develop vascular instability in association with severe hepatic and systemic injury [4,26], West Nile virus predominantly affects specialized neurovascular interfaces [22], and severe Zika disease follows another predominantly neurological and immunological trajectory [4,23,24]. Similar downstream vascular abnormalities therefore should not be interpreted as evidence of identical immune kinetics, pathogen persistence, or hemodynamic physiology.

The SIMVAC hypothesis is consequently falsifiable. Validation would require pathogen-confirmed and clinically stratified cohorts, appropriate comparator groups, serial rather than isolated biomarker measurements, predefined clinical endpoints, and standardized assays. Failure to identify a reproducible integrated immunovascular signature across such cohorts would require modification or rejection of the proposed framework.

## 6. Pathogen-Specific Evidence for Immunovascular Convergence

Dengue provides the strongest mechanistic anchor for evaluating whether severe arboviral infections can converge with sepsis on a shared immunovascular phenotype. Acute vascular permeability was recognized decades ago as a defining physiological abnormality of severe dengue, establishing plasma leakage rather than hemorrhage alone as a principal determinant of circulatory compromise [9]. Subsequent experimental studies provided molecular resolution by identifying dengue virus non-structural protein 1 (NS1) as a direct mediator of endothelial barrier dysfunction. NS1 can promote glycocalyx degradation and activate Toll-like receptor 4–dependent inflammatory signaling without requiring productive viral replication within endothelial cells [6,10,11]. This produces a biologically coherent sequence from a pathogen-specific determinant to endothelial destabilization, increased permeability, intravascular volume redistribution, and shock [7,52]. Sequential infection with heterologous dengue serotypes adds an immunological amplification pathway through antibody-dependent enhancement, which can increase viral burden and intensify downstream inflammatory responses [53]. Dengue therefore represents the best-supported proof of concept that an arboviral infection can reach a critical immunovascular state through identifiable viral and host-dependent mechanisms [10,11,14,15].

Zika virus illustrates a different and substantially less established trajectory. Most infections are asymptomatic or clinically mild, and severe systemic presentations constitute a minority [4,23,24]. Nevertheless, reported complications include immune-mediated thrombocytopenia, neurological disease, encephalitis, Guillain–Barré syndrome. These observations demonstrate that clinically important vascular and inflammatory dysfunction is not exclusive to dengue, but they do not establish that Zika follows an equivalent molecular pathway. Its marked neurotropism further distinguishes its biological trajectory [23,24]. When systemic deterioration occurs, interactions among viral burden, innate immune activation, endothelial responses, and host susceptibility may generate a septic-like phenotype, but the relative contributions of these mechanisms remain incompletely defined. Zika should therefore be positioned within SIMVAC as a biologically plausible but less mechanistically resolved model rather than as evidence of universal arboviral endotheliopathy [35,54,55].

Yellow fever provides another distinct route toward systemic vascular dysfunction. Severe disease is characterized by hepatocellular injury, metabolic dysfunction, coagulopathy, systemic inflammation, and progressive multiorgan involvement [4,26]. Endothelial dysfunction has also been associated with disease severity, including increased circulating NS1 and syndecan-1 [26]. Thus, unlike dengue, in which direct NS1-mediated disruption of endothelial barrier integrity is comparatively well characterized, yellow fever may approach vascular instability through the interaction of severe hepatic injury, systemic inflammation, coagulation abnormalities, and endothelial dysfunction [4,26]. Yellow fever therefore represents an organ-driven route toward the broader immunovascular phenotype rather than a dengue-like mechanistic pathway.

West Nile virus further demonstrates that endothelial dysfunction can be anatomically specialized. Its most severe manifestations are neuroinvasive, including meningitis, encephalitis, and acute flaccid paralysis [22,56]. Viral access to the central nervous system can involve interactions with the blood–brain barrier, including infection of brain microvascular endothelial cells and modulation of tight-junction and cell-adhesion pathways [57]. Thus, the relevant vascular lesion does not necessarily require generalized capillary leakage; disruption of an organ-specific endothelial interface can contribute to severe disease while preserving the principle that endothelial vulnerability represents an important interface between viral infection and organ dysfunction [57].

The evidence becomes considerably weaker for emerging arboviruses such as Mayaro, Oropouche, Venezuelan equine encephalitis, and Rocio viruses. Mayaro and Oropouche viruses can produce clinically important systemic disease and have been associated with complications and occasional fatal outcomes [58]; Venezuelan equine encephalitis virus can cause febrile and neuroinvasive disease [59]; and Rocio virus was identified as the etiologic agent of a major encephalitis outbreak in Brazil, including fatal cases [60]. However, direct mechanistic evidence linking these viruses to glycocalyx degradation, Ang-2/Tie2 dysregulation, S1P depletion, complement-mediated endothelial injury, NETosis, or vWF/ADAMTS13 imbalance remains limited or absent. These viruses should therefore not be positioned at the same evidentiary level as dengue and instead represent a testable frontier of the SIMVAC hypothesis [58,59,60].

Across these pathogen-specific trajectories, clinical convergence may nevertheless emerge when endothelial integrity can no longer be maintained. Glycocalyx disruption and imbalance between destabilizing and protective vascular signals, including Ang-2/Tie2 and sphingosine-1-phosphate pathways, can promote increased permeability and effective intravascular volume loss. Complement activation can amplify endothelial and leukocyte responses, while excessive NET formation can connect innate immunity to platelet activation and microvascular thrombosis. Simultaneously, disproportionate von Willebrand factor release relative to ADAMTS13 activity can favor platelet consumption and thrombotic microangiopathic features. These mechanisms can generate overlapping manifestations such as hypotension, tachycardia, thrombocytopenia, altered tissue perfusion, lactate elevation, and progressive organ dysfunction [30,33,34,45].

**Figure 1:**
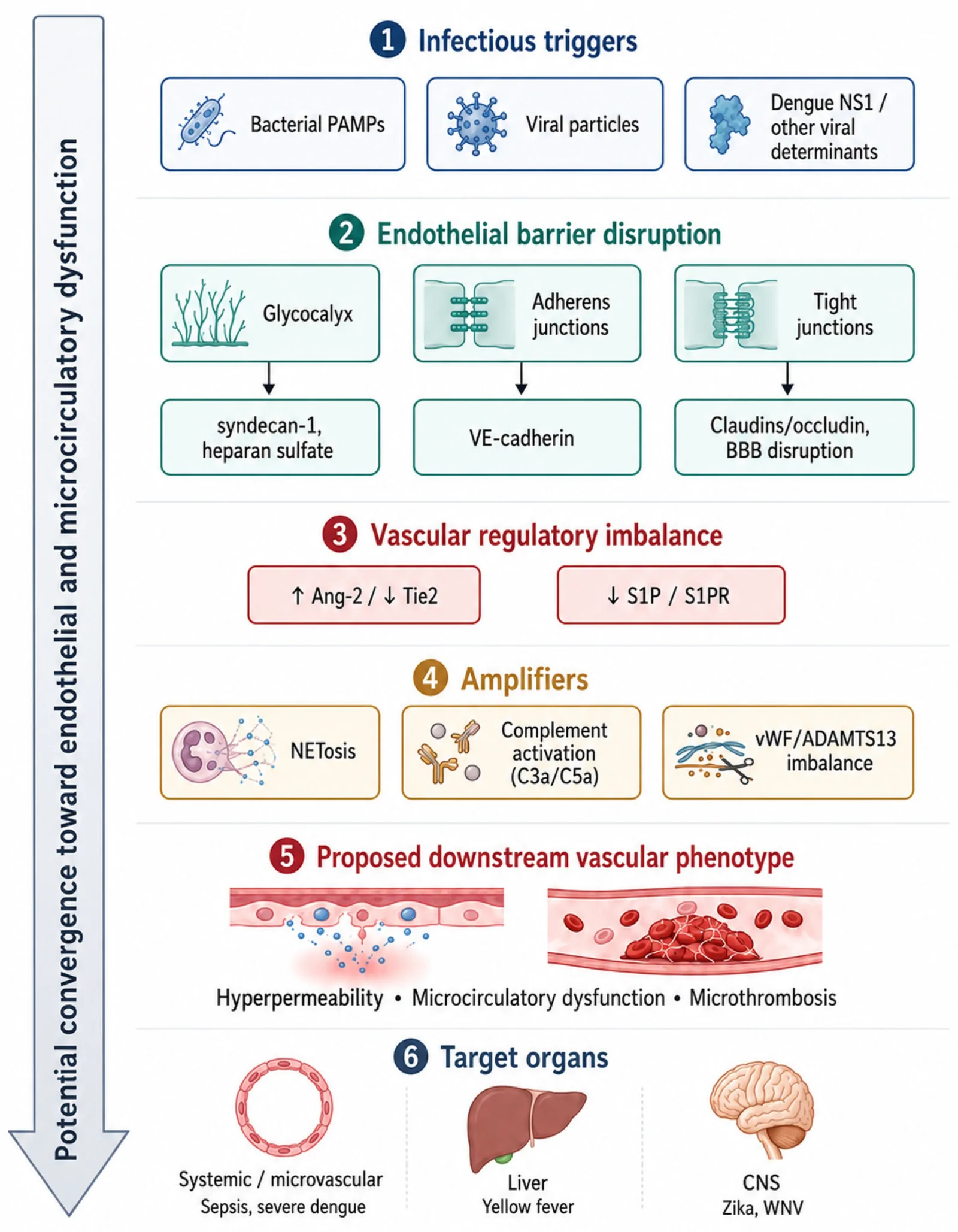
Proposed mechanistic architecture of the SIMVAC framework. Pathogen-specific infectious triggers may disrupt endothelial barrier integrity and vascular regulatory pathways, while complement activation, NETosis, and vWF/ADAMTS13 imbalance may amplify downstream vascular dysfunction. Potential functional convergence includes hyperpermeability, microcirculatory impairment, microthrombosis, and organ injury. The framework does not imply mechanistic equivalence across pathogens; evidence is strongest for bacterial sepsis and dengue and remains substantially more limited for other arboviruses. Upward (↑) and downward (↓) arrows indicate increased and decreased levels or signaling activity, respectively, relative to physiological homeostasis.

The proposed SIMVAC framework therefore does not require that dengue, Zika, yellow fever, West Nile virus infection, emerging arboviroses, and bacterial sepsis share a single molecular pathway. Its biological basis lies instead in functional convergence after different initiating injuries. Dengue reaches this state predominantly through well-characterized viral–endothelial interactions; yellow fever may approach it through severe hepatic and systemic injury; West Nile virus demonstrates organ-specific endothelial vulnerability; and evidence for emerging arboviruses remains hypothesis-generating. SIMVAC should consequently be understood as a stratified mechanistic framework in which distinct infectious processes are evaluated according to the strength of evidence that they converge on endothelial destabilization, altered permeability, immunothrombosis, microcirculatory failure, and organ dysfunction [30,33,34,45]. This proposed architecture of pathogen-specific injury and downstream functional convergence is summarized in Figure 1. The relative strength and nature of the evidence supporting SIMVAC-relevant immunovascular convergence across individual pathogens are summarized in Table 1. Because SIMVAC is not a validated diagnostic or therapeutic construct, its potential clinical relevance should remain anchored in conventional etiological assessment, bedside severity evaluation, and evidence-based supportive management; a pragmatic evaluation framework for endemic settings is provided in Table 2.

## 7. Discussion

The principal contribution of the SIMVAC framework is not the assertion that bacterial sepsis and severe arboviral infections share a common pathogenesis, but the narrower hypothesis that etiologically distinct infections may converge on selected components of endothelial and microcirculatory dysfunction [7,10,26,27,28,29,37,48]. The available evidence is markedly asymmetric. Dengue provides the strongest arboviral mechanistic anchor because decades of physiological observations demonstrating plasma leakage [9] have subsequently been connected with experimentally defined effects of NS1 on endothelial barrier integrity and the glycocalyx [10,11]. This level of mechanistic resolution is not currently available for most other arboviruses [22,23,24,25,26].

Preserving pathogen-specific differences is therefore essential. Zika virus is characterized principally by neurotropism, placental biology, and uncommon neurological or severe systemic complications [4,23,24]; yellow fever follows a trajectory dominated by hepatocellular injury, metabolic dysfunction, and coagulopathy [4,26]; West Nile virus predominantly affects specialized neurovascular interfaces [22]; and chikungunya is primarily arthritogenic [4,25]. Evidence for Mayaro, Oropouche, Venezuelan equine encephalitis, and Rocio viruses remains considerably more limited. Similar manifestations such as hypotension, thrombocytopenia, altered perfusion, vascular permeability, or organ dysfunction should consequently be interpreted as potentially convergent downstream states rather than evidence of identical upstream mechanisms [10,22,23,24,25,26].

The same distinction applies when comparing arboviral disease with bacterial sepsis. Bacterial sepsis may involve profound vascular dysregulation, endothelial activation, coagulation abnormalities, and heterogeneous microcirculatory impairment [5,7]. Arboviral infections follow different temporal architectures determined by viral replication, viremia, tissue tropism, pre-existing immunity, and pathogen-specific host responses [9,10,11,22,24]. Severe dengue, in particular, may develop clinically important plasma leakage during a relatively defined critical phase [4,10,11]. SIMVAC therefore does not define these hemodynamic or immunological trajectories as equivalent; it provides a framework for testing whether selected endothelial functions become similarly disrupted despite those differences.

Biomarkers proposed within this framework should currently be considered investigational. Syndecan-1 [7,27], angiopoietin-2 [37], sphingosine-1-phosphate [7], complement mediators [29], NET-associated markers [28], von Willebrand factor/ADAMTS13 [48], IL-6 and VEGF [37], and ST2-related signaling [62] provide mechanistic or translational information regarding endothelial and inflammatory biology, but their sensitivity, specificity, optimal sampling windows, clinically meaningful thresholds, and incremental value for a SIMVAC construct have not been established. Their clinical implementation is additionally constrained by assay availability, analytical standardization, turnaround time, and cost, particularly in resource-limited endemic settings. At present, no minimal SIMVAC biomarker panel can therefore be recommended. Conventional assessment—including serial hematocrit, platelet count, hemodynamic evaluation, capillary refill, urine output, lactate when available, evidence of plasma leakage, and organ dysfunction—should remain the clinically actionable foundation, complemented by pathogen-directed microbiological or molecular testing [4].

The SIMVAC framework likewise should not determine empirical antimicrobial therapy. Patients presenting with possible bacterial sepsis require timely management according to the initial probability of bacterial infection [5], while doxycycline should be considered only when clinical and epidemiological features maintain rickettsial disease as a meaningful alternative or coinfection [61,63]. Etiology should be repeatedly reassessed as microbiological, molecular, imaging, epidemiological, and clinical information becomes available [5]. Confirmation of an arboviral infection does not exclude bacterial coinfection; however, empirical antibacterial treatment should be de-escalated or discontinued when bacterial infection is no longer supported and the clinical trajectory is adequately explained by the confirmed alternative diagnosis [5].

Future evaluation of SIMVAC should therefore move from descriptive similarity toward prospective measurement. Pathogen-confirmed cohorts should incorporate predefined clinical endpoints, serial assessment of endothelial and hemostatic biomarkers, appropriate bacterial and viral comparator groups, standardized timing of sample collection, and clinically relevant measures of microcirculatory and organ dysfunction. Such studies should determine not only whether an integrated immunovascular signature can be reproduced, but also whether it adds prognostic or discriminatory information beyond pathogen identification and conventional severity assessment. Failure to demonstrate such reproducible convergence would constitute evidence against the current formulation of the framework and should lead to its modification or rejection.

### Limitations

This review has limitations inherent to its narrative and hypothesis-generating design. Because the literature was not selected through a protocolized systematic-review methodology, relevant studies may have been missed and selection bias cannot be excluded. The available evidence is also markedly heterogeneous in study design, population, diagnostic methods, experimental systems, biomarker assessment, disease stage, and outcome definitions, limiting direct comparisons across pathogens. Importantly, the evidentiary base is highly asymmetric: dengue and bacterial sepsis have substantially greater mechanistic and translational support than most other arboviral infections, and evidence derived from these conditions should not be extrapolated to less-characterized pathogens. The SIMVAC framework has no prospectively validated operational definition, diagnostic thresholds, sensitivity or specificity estimates, or validated biomarker panel. In addition, the availability, cost, turnaround time, and analytical standardization of several proposed biomarkers may limit their applicability in resource-constrained endemic settings. SIMVAC should therefore be interpreted as a testable conceptual framework whose validity, clinical relevance, and boundaries require prospective evaluation rather than as an established diagnostic or therapeutic entity.

## 8. Conclusions

The SIMVAC framework proposes that etiologically distinct severe infections may converge on selected components of endothelial, inflammatory, hemostatic, and microcirculatory dysfunction without sharing identical upstream mechanisms. Dengue currently provides the strongest arboviral mechanistic proof of concept, whereas evidence for Zika, yellow fever, West Nile virus, chikungunya, and particularly Mayaro, Oropouche, Venezuelan equine encephalitis, and Rocio viruses remains heterogeneous and substantially less complete.

SIMVAC should therefore be regarded as a hypothesis-generating and falsifiable model rather than a validated syndrome, diagnostic tool, or biomarker-defined clinical entity. Its value now depends on prospective testing in pathogen-confirmed, clinically stratified cohorts using predefined endpoints, serial measurements, appropriate comparator groups, and validated biomarker assays. Such studies should establish whether a reproducible integrated immunovascular state exists across distinct severe infections and whether its measurement provides information beyond conventional severity assessment and etiological diagnosis.

## Figures and Tables

**Table 1:** Pathogen-Specific Strength and Nature of Evidence for SIMVAC-Relevant Immunovascular Convergence.

Infection	Main SIMVAC-Relevant Pathway	Principal Evidence Base	Strength of Evidence for Vascular Convergence	Interpretation Within SIMVAC
Bacterial sepsis [1,5,7,8,33]	Endothelial activation, glycocalyx injury, vasoplegia, coagulation–complement crosstalk, microcirculatory dysfunction	Human clinical/translational studies and experimental models	Strong	Reference comparator for systemic immunovascular dysfunction; not mechanistically equivalent to arboviral disease
Dengue [6,9,10,11,14,15,16,27,28,29,41,48,53]	NS1-associated endothelial barrier disruption, glycocalyx degradation, plasma leakage, immune and hemostatic amplification	Historical physiological studies, human translational studies, experimental models	Strongest arboviral evidence	Principal arboviral mechanistic proof of concept
Zika [4,23,24]	Neurovascular and placental involvement, thrombocytopenia, and uncommon severe systemic complications	Clinical observations and experimental evidence	Limited–moderate; context specific	Biologically plausible but substantially less resolved than dengue
Yellow fever [4,26]	Severe hepatocellular injury, coagulopathy, systemic inflammation and secondary vascular instability	Clinical/pathological observations with indirect mechanistic support	Moderate for systemic injury; limited for direct endothelial mechanisms	Organ-driven route toward vascular dysfunction rather than a dengue-like pathway
West Nile virus [22,56,57]	Blood–brain barrier dysfunction and organ-specific endothelial injury	Clinical and experimental neurovascular evidence	Moderate for neurovascular involvement	Supports organ-specific endothelial vulnerability, not generalized capillary leakage
Chikungunya [4,25]	Predominantly inflammatory and musculoskeletal disease; systemic vascular involvement incompletely characterized	Primarily clinical evidence	Limited for SIMVAC-relevant systemic convergence	Should not be assumed to share dengue-like endothelial mechanisms
Mayaro, Oropouche, Venezuelan equine encephalitis, and Rocio viruses [58,59]	Severe febrile, neurological, inflammatory, or systemic manifestations; direct SIMVAC-relevant endothelial mechanisms remain unestablished	Predominantly descriptive clinical/epidemiological evidence	Limited/hypothesis-generating	Testable frontier of the framework; direct SIMVAC-relevant mechanisms remain unestablished

**Table 2:** Pragmatic evaluation framework for severe arboviral disease–sepsis overlap in endemic settings.

Domain	Assessment	Interpretation/Clinical Action
Initial presentation	Fever with hypotension, altered tissue perfusion, organ dysfunction, thrombocytopenia and/or evidence of plasma leakage	Consider both bacterial sepsis and severe arboviral disease; SIMVAC should not replace etiological assessment
Epidemiological context	Vector exposure, outbreak activity, travel, geographic setting, potential bacterial or rickettsial exposure	Adjust pre-test probability and prioritize pathogen-directed testing
Disease phase and etiological testing	Clinical timeline; dengue NS1 antigen, RT-PCR, serology or other pathogen-directed testing as appropriate	Interpret virological tests according to disease phase and suspected pathogen
Conventional bedside assessment	Serial hematocrit, platelet count, lactate when available, capillary refill, urine output, hemodynamics, evidence of plasma leakage and organ dysfunction	Remains the principal clinically available assessment of disease severity and circulatory deterioration
Candidate investigational immunovascular biomarkers	Syndecan-1, Ang-2, S1P, complement mediators, NET-associated markers, vWF/ADAMTS13, IL-6, ST2, and VEGF	Research/translational use; no validated SIMVAC-specific thresholds, sensitivity/specificity estimates, or bedside panel currently exist
Hemodynamic management	Dynamic reassessment of perfusion and fluid responsiveness	Individualize fluid administration; avoid unnecessary fluid overload; use vasopressors when clinically indicated; do not use anticoagulation solely on the basis of the SIMVAC framework
Antimicrobial stewardship	Repeated reassessment using microbiological, molecular, imaging, epidemiological and clinical information	Empirical antibacterial therapy should reflect the probability of bacterial infection or coinfection. Doxycycline should be considered only when rickettsial infection remains clinically and epidemiologically plausible. De-escalate or discontinue empirical antibacterials when an alternative arboviral diagnosis is established and bacterial infection is no longer supported, unless another indication exists
Organ-specific complications	Neurological, hepatic, cardiac, renal and hematological monitoring according to pathogen and clinical trajectory	Recognize pathogen-specific complications rather than assuming a uniform SIMVAC presentation

Note: This table does not constitute a validated SIMVAC diagnostic or treatment algorithm. Clinical management recommendations are based on current WHO guidance for arboviral diseases [4], the Surviving Sepsis Campaign [5], and rickettsial disease guidance [61]. Candidate immunovascular biomarkers remain investigational.

## Data Availability

No new data were created or analyzed in this study. Data sharing is not applicable to this article.
